# Comparison Between Conventional and Variable-Angle Locking Compression Plates in Complex Proximal Tibia Fractures

**DOI:** 10.7759/cureus.69237

**Published:** 2024-09-11

**Authors:** Prashant P Singh, Sunil Kumar, Dinesh Kumar, Pradeep K Gupta, Sanjeev Joshi, Rajeev Kumar, Harish Kumar, Gaya Deen

**Affiliations:** 1 Orthopedics, Uttar Pradesh University of Medical Sciences Saifai, Etawah, IND

**Keywords:** conventional fixed angled proximal tibia locking plate, oxford knee scores, rasmussen functional and radiological grading system, schatzker’s classification, tibial plateau fracture, variable angle locking plate (valcp)

## Abstract

Background: Proximal tibial fractures, particularly those involving the tibial plateau, are complex injuries that require careful management to restore knee function and prevent long-term disability. Recent advancements have introduced variable-angle locking compression plates (VALCP) as a potential alternative to the widely used fixed-angle locking plating techniques. These plates allow for more precise screw placement, potentially improving fixation and clinical outcomes. The goal of this study was to find out how well conventional locking plates and VALCP treat Schatzker's type I, II, and III tibial plateau fractures in terms of clinical, functional, and radiological outcomes. We evaluated the outcomes using the Rasmussen functional and radiological grading systems, as well as the Oxford Knee Score (OKS).

Methods: This prospective study was undertaken by a tertiary care medical institute from January 2020 to August 2021. The study included a total of 60 patients with Schatzker's type I, II, and III tibial plateau fractures. These patients were randomly assigned to two groups, with each group consisting of 30 patients. Conventional locking compression plates (CLCP) treated one group, while VALCP treated the other. Clinical, functional, and radiological outcomes of patients were evaluated using the OKS, Rasmussen's functional grading system, and Rasmussen's radiological grading system. Additionally, the study documented and examined the duration of the surgical procedure, the stability of the fixation, and any complications that occurred in the postoperative phase over a span of six months.

Results: The study included 52 males and eight females, aged 19 to 65 years. The mean age was 42.66 years for the conventional LCP group and 35.6 years for the VALCP group. Road traffic accidents were the most common cause of injury, with 86.67% in the VALCP group and 70% in the conventional group. In both groups, the majority of fractures were Schatzker type II. At the six-month follow-up, 60% of VALCP patients had excellent functional outcomes compared to 50% in the conventional group. Radiologically, 80% of VALCP patients had excellent results versus 73.33% in the conventional group. The OKS showed that 86.67% of VALCP patients had excellent results, compared to 73.33% in the conventional group. While VALCP showed slightly better outcomes, the differences were not statistically significant. Complications were minimal, with 90% of VALCP and 86.67% of conventional LCP patients experiencing no complications.

Conclusion: The small number of patients, short-term study, and heterogeneity of fractures constitute a limitation of this study. VALCP plating in tibial plateau fractures is a good treatment modality because it seems to improve fixation, provides early mobilization, and has excellent to good functional and radiological outcomes. However, no significant difference in functional and radiological outcomes was found between the conventional and VALCP groups.

## Introduction

Proximal tibial fractures are among the most common types of tibial fractures [1]. Unicondylar tibial plateau fractures (Schatzker types I, II, III, IV) account for approximately 64.5% of these injuries, while bicondylar fractures (types V and VI) represent 35.8% [2]. Most tibial plateau fractures result from high-energy trauma, typically involving a valgus (more common) or varus force, along with indirect shear stresses [3,4]. The majority of these fractures are intra-articular. In older patients with osteopenic bone, depression-type fractures are more common due to the reduced ability of their subchondral bone to withstand axially directed loads [5].

Surgical treatment is generally required for these fractures, with the primary goals being to restore congruent articular surfaces of the tibial condyles, maintain the mechanical axis, and restore ligamentous stability. The aim is to achieve a solid union of the fracture, resulting in a painless, mobile, and stable knee joint [6,7].

Considering the challenges posed by soft tissue concerns and complex fracture patterns, advancements in fracture fixation have led to the development of biological fixation techniques using plates, known as minimally invasive plate osteosynthesis (MIPO). MIPO preserves the periosteum and maintains the biological environment of the fracture and surrounding bone, leading to the creation of internal fixators [8]. As the understanding of biological fixation has improved, new plate designs have emerged, including the less invasive stabilizing system (LISS). Research aimed at combining these techniques resulted in the AO locking compression plate (LCP) [9].

The initial generation of angular stable locking plates had limitations, particularly in achieving optimal screw placement within the bone fragment due to their pre-shaped design and incorporated threads. There was a risk of screw misplacement or fixation in areas with poor bone quality, potentially leading to secondary reduction loss or screw loosening. To address these challenges, the concept of polyaxial (variable angle) plate osteosynthesis was developed, a technique already in use in spinal surgery. The 3.5 mm variable-angle locking compression plates (VALCP) Proximal Tibia Plate (DePuy Synthes, USA), with variable-angle locking screws, allows surgeons to create a fixed-angle construct while also providing the flexibility to choose the screw trajectory before locking it in place. This fixed-angle construct is particularly advantageous in osteopenic bone or multifragmentary bridge-plated fractures, where screw fixation does not rely on plate-to-bone compression to bear the patient's load [10].

Although numerous studies have explored the functional outcomes of proximal tibia fractures treated with conventional (fixed-angle) and variable-angle locking plates, there is a lack of comparative studies between these two methods. This study was conducted to evaluate the comparative functional outcomes of variable-angle locking plates versus conventional (fixed-angle) locking plates in the treatment of proximal tibia fractures.

## Materials and methods

All patients admitted to the orthopedics department of Uttar Pradesh University of Medical Sciences Saifai, Etawah, India, with tibial plateau fractures (Schatzker types I to III) who met the inclusion criteria between January 2020 and August 2021 were considered for this prospective interventional study. Ethical clearance was obtained from the Ethical Committee Uttar Pradesh University of Medical Sciences Saifai, India, and written informed consent was acquired from all patients or their families prior to their participation in the study. The study included skeletally mature patients with tibial plateau fractures classified as Schatzker types I, II, and III. The study did not include tibial plateau fractures involving the medial condyle (Schatzker type IV), bicondylar fractures (Schatzker types V and VI), or tibial plateau fractures accompanied by fractures in other bones within the same limb. Patients with injuries older than three weeks, pathological fractures, open fractures (Gustilo-Anderson grade III fractures), preexisting joint conditions like osteoarthritis, inflammatory arthritis, and severe systemic illnesses were also excluded from the study. Initially, 70 patients were enrolled in the study; however, 10 were lost to follow-up, resulting in a final sample of 60 patients.

Patients were evaluated based on their medical history and the mechanism of injury. The injured lower limb was immobilized using an above-knee splint, and anteroposterior, lateral, and oblique X-rays were taken. Upon admission, calcaneal pin traction was applied, and the limb was positioned on a Bohler Braun splint to ensure proper alignment of the fractured fragments and allow for soft tissue healing. Adequate time was provided for soft tissue recovery, typically between 5 and 14 days. If necessary, three-dimensional computed tomography (3D CT) was performed to further assess the fracture pattern. Basic and routine investigations of all cases were done and preanesthetic surgical fitness was obtained.

Patients were randomly assigned to one of two groups using simple computer randomization. Those in the VALCP group were treated with a variable-angle locking plate, while those in the CLCP group received treatment with a conventional fixed-angle proximal tibia locking plate.

Implants

**Figure 1:**
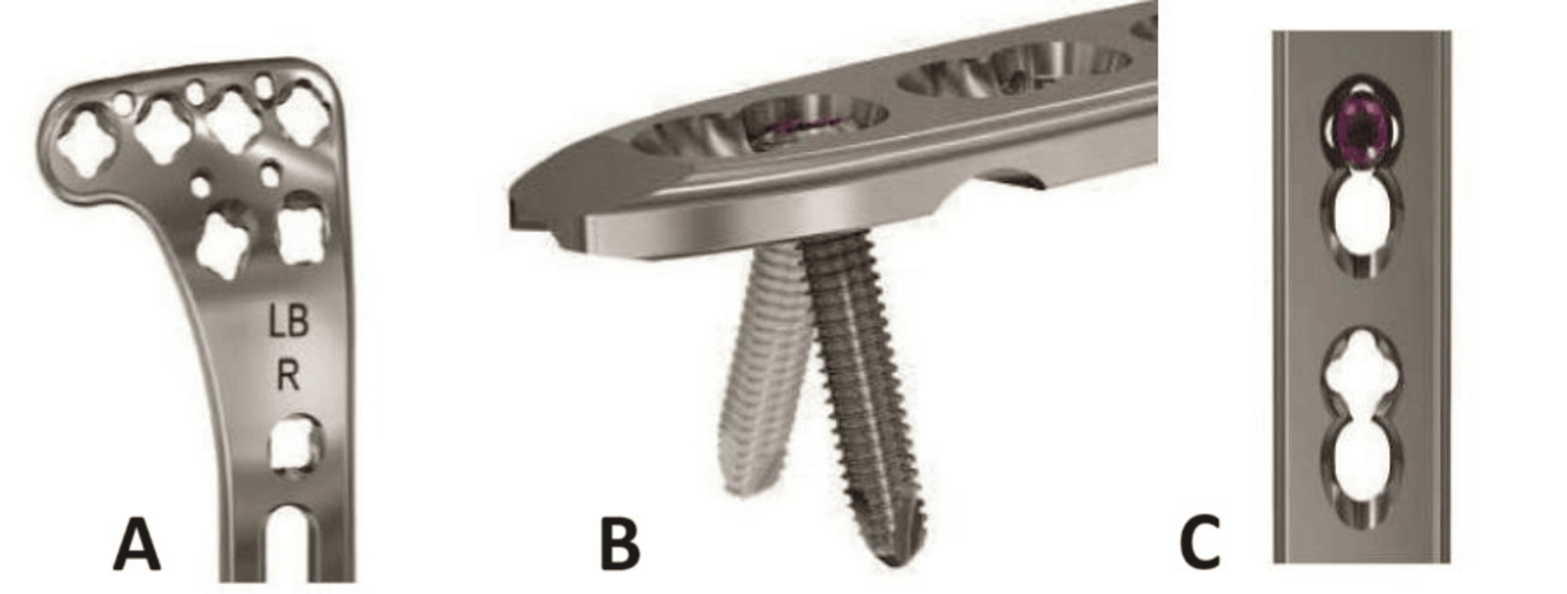
3.5 mm variable-angle locking plate A) Variable angle proximal tibia locking plate right side; B) Variable angle holes of the plate allow a screw angulation of 15° in each direction forming a 30° cone around the central axis of the plate hole; C) 3.5 mm variable angle locking screws are color coded for easy differentiation from standard locking screws

**Figure 2:**
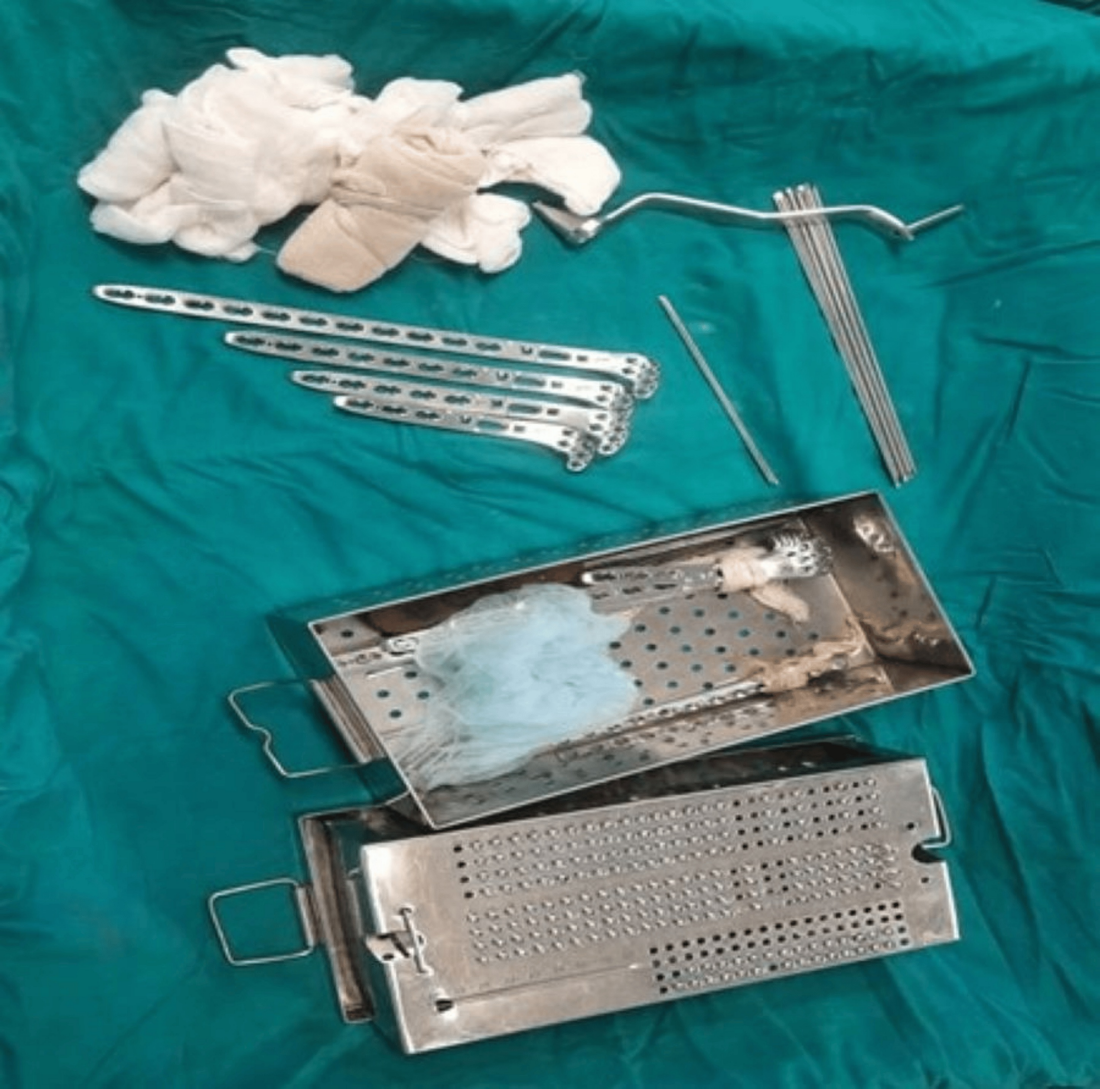
Variable angle proximal tibia locking plates and locking screws set with instruments for application of plates and screws

VALCP Proximal Tibia Plate is part of the VALCP Periarticular Plating System which merges variable angle locking screw technology with conventional plating techniques. The variable angle locking allows a screw angulation of 15° in each direction forming a 30° cone around the central axis of the plate hole. The 3.5 mm VALCP Proximal Tibia Plate has variable angle holes in the plate head and neck, along with variable-angle combi holes in the plate shaft that combine a dynamic compression unit (DCU) hole with a variable angle locking screw hole. The variable-angle combi hole provides the flexibility of axial compression and variable angle locking capability throughout the length of the plate shaft (Figures 1, 2).

Conventional Fixed Angle Locking Compression Plate

**Figure 3:**
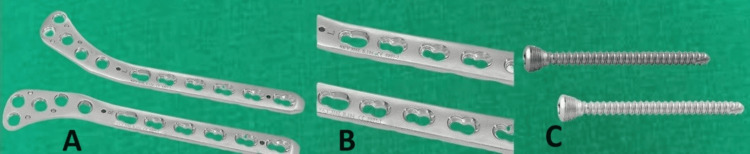
Conventional 4.5 mm fixed angle proximal tibia locking plate with locking screw A) proximal tibia locking plates right and left side; B) Combi holes in plate; C) Locking screws of plate

These implants combine the principles of angular stable construct and compression plating. Its design and characteristics allow it to be used as a minimally invasive approach by using the principles of biological osteosynthesis (Figure 3).

**Figure 4:**
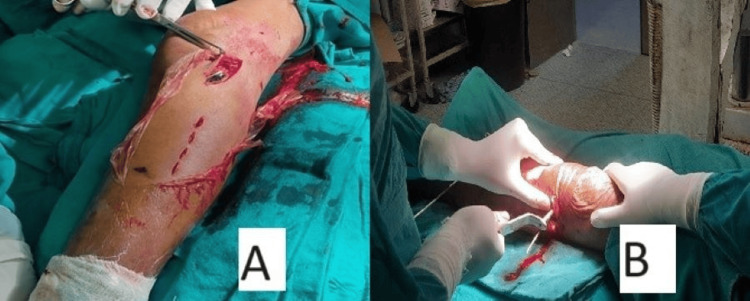
Proximal tibia plate fixation with minimally invasive plate osteosynthesis (MIPO) A) Proximal tibia locking plate application with lateral minimal incision and screws placement through stab incisions; B) Drilling of bone for proximal locking screws placement in plate

All surgeries were performed by a single senior orthopedic surgeon using a lateral MIPO approach to the proximal tibia. For complex intra-articular fractures, an open anterolateral approach was used. The articular fracture fragments were directly reduced, with the reduction confirmed using image intensification and, when possible, direct visualization. If there was a depression in the articular surface, it was elevated and bone grafting was performed (Figure 4).

In the VALCP group, screw insertion in the plate head begins with the fixed-angle screws in the proximal row, followed by the insertion of variable-angle screws, which are placed around the fixed-angle screws. In contrast, the conventional LCP group involves using a LCP along with 4.5 mm cortical, locking, and cancellous screws of varying lengths applied.

In all the cases we placed a drain in the wound and closed it in layers. On the third postoperative day, the drain was removed, and stitches were removed on the 12 postoperative day. We initiated progressive muscle-strengthening exercises and passive exercises within 24 hours after surgery in all cases. For a minimum of 10 weeks, we kept the patient off all weights. Partial weight-bearing began between 10 and 14 weeks, depending on the fracture configuration and X-ray correlation. Only after radiological confirmation of fracture healing did we allow full weight-bearing. We followed up and examined the patient weekly for one month after discharge, then again every three months, and finally at six months. We assessed the clinical outcomes of the two groups using standardized questionnaires, with a focus on a range of motion and weight-bearing ability. We assessed the functional outcomes of each group at each follow-up using the Oxford Knee Score (OKS) and Rasmussen's functional grading system. We used Rasmussen's radiological grading system for radiological evaluation.

At each follow-up, we utilized the OKS as a patient-reported outcome measure to evaluate knee function and discomfort. The assessment comprises 12 components, each specifically targeting various facets of knee functionality and the repercussions of knee issues on everyday activities. We assigned a numerical value ranging from 0 to 4 to each item and then added up the scores for all 12 things, giving a total score that can vary from 0 to 48. Results ranging from 0 to 19 were categorized as poor, while scores between 20 and 29 were considered fair. A good result fell within the range of 30 to 39, and an excellent result was achieved with scores between 40 and 48.

In addition, we employed Rasmussen's Functional Grading System in conjunction with the Oxford Knee Scoring System to assess their clinical outcomes. The assessment measures the severity of knee discomfort, the individual's capacity to walk, the range of motion of the knee joint, and the general stability of the knee. We evaluated each category separately and determined the functional outcome by considering the overall score. The grading parameters consist of pain (rated on a scale of 0-6), walking ability (rated on a scale of 0-6), knee extension (rated on a scale of 0-6), and stability (rated on a scale of 0-6). The cumulative score spans from 0 to 30. The functional outcomes were classified as follows: excellent (27-30 points), good (20-26 points), fair (10-19 points), and poor (0-9 points).

We assessed the radiological results of cases utilizing Rasmussen's Radiological Grading System. The main purpose of this assessment is to evaluate the anatomical alignment of the joint surface, the positioning of the tibial plateau, and the occurrence of any arthritic changes resulting from a traumatic event. The characteristics consist of articular depression (rated on a scale of 0-6), condyle widening (rated on a scale of 0-6), and varus/valgus angulation (rated on a scale of 0-6). The total score ranges from 0 to 18, with the following classifications: excellent (18 points), good (12-17 points), fair (6-11 points), and poor (0-5 points).

Statistical analysis

The collected data were statistically analyzed using IBM SPSS Statistics for Windows, Version 24 (Released 2016; IBM Corp., Armonk, New York, United States). Numerical variables were presented as mean and standard deviation, while categorical variables were expressed as frequency and percentage. The t-test was used to compare the means of various parameters, with two-tailed p-values calculated and 95% confidence intervals determined using the normal approximation method. For paired categorical variables, Fischer's exact test and chi-square test were used. A p-value of less than 0.05 was considered statistically significant.

## Results

The study included 60 patients, with 30 assigned to the variable-angle LCP group and 30 to the traditional LCP group. The test group consisted of 52 males and eight females, with all patients receiving follow-up care for at least six months. Clinical and radiological assessments were conducted using the OKS, Rasmussen functional grading system, and Rasmussen radiological grading system.

Patients' ages ranged from 19 to 65 years, with a mean age of 42.66 years for those in the conventional LCP group and 35.6 years for those in the variable-angle LCP group. In the VALCP group, 26 patients (86.67%) were male and four (13.33%) were female; 19 patients (63.33%) had a right-side fracture, and 11 patients (36.67%) had a left-side fracture. Conversely, in the conventional LCP group, 17 patients (56.67%) had a right-side fracture, and 13 patients (43.33%) had a left-side fracture. Road traffic accident (RTA) was the primary mode of injury, with 26 patients (86.67%) in the VALCP group and 21 patients (70%) in the conventional LCP group sustaining injuries this way. Most cases in both groups were Schetzker's type II fractures, with four patients (13.33%) having type I, 20 patients (66.67%) having type II, and six patients (20%) having type III fractures in the VALCP group, compared to five patients (16.66%) with type I, 16 patients (53.33%) with type II, and nine patients (30%) with type III fractures in the conventional LCP group. No significant difference was observed in blood loss between the two groups (Table 1).

**Table 1:** Study demographics Numerical variables expressed as mean±SD, values of two groups were compared using unpaired student t-test. The chi-square test was used to compare groups for categorical variables

Variables	CLCP group			VALCP group			p-value
Age	No.	Percentage	Mean±SD	No.	Percentage	Mean±SD	
<40 years	8	26.67%	42.66±7.77	22	73.33%	35.65±11.4	0.002
40-50 years	19	26.67%		4	13.33%		
>50 years	3	10.00%		4	13.33%		
Sex							0.036
Male	19	63.33%		26	86.67%		
Female	11	36.67%		4	13.33%		
Mode of injury							
Fall from height	8	26.67%		3	10.00%		0.246
Trivial trauma	1	3.33%		1	3.33%		
RTA	21	70.0%		26	86.67%		
Side Involve							0.598
Right side	13	43.33%		11	36.67%		
Left side	17	56.67%		19	63.33%		
Fracture type (Schatzker’s)							
Type I	5	16.66%		4	13.33%		0.136
Type II	16	53.33%		20	66.67%		
Type III	9	30.00%		6	20.00%		
Mean duration of surgery (min)			83.17±7.71			80.67±7.28	0.2017

In the variable angle plating group, 27 patients (90%) experienced no complications; however, one patient (3.33%) developed a superficial infection, one patient (3.33%) had a varus deformity due to medial collapse, and one patient (3.33%) suffered a deep infection necessitating implant removal. In the conventional plating group, 26 patients (86.67%) had no complications; two patients (6.67%) developed superficial infections, and one patient (3.33%) had a deep infection (Table 2).

**Table 2:** Complications of both groups LCP: locking compression plate

Complications	Conventional LCP		Variable LCP	
	No.	Percentage (%)	No.	Percentage (%)
Deep infection and removal of implant	1	3.33	1	3.33
Superficial infection	2	6.67	1	3.33
Varus	1	3.33	1	3.33
Nill	26	86.67	27	90.00
Total	30	100.00	30	100.00

In the VALCP group, patients began partial weight-bearing at an average of 13.4 weeks, with a range from 10 to 20 weeks. They started full weight-bearing at an average of 18.93 weeks, with a minimum of 14 weeks and a maximum of 30 weeks. For the conventional LCP group, partial weight-bearing began at an average of 14 weeks, with the same range of 10 to 20 weeks, while full weight-bearing started at an average of 19.73 weeks, ranging from 14 to 28 weeks (Tables 3-4).

**Table 3:** Partial weight bearing in both groups LCP: locking compression plate

Partial weight bearing (weeks)				
Weeks	Conventional LCP		Variable LCP	
	No.	Percentage (%)	No.	Percentage (%)
10	2	6.67	2	6.67
12	12	40.00	15	50.00
14	8	26.67	8	26.67
16	2	6.67	1	3.33
18	4	13.33	3	10.00
20	2	6.67	1	3.33

**Table 4:** Complete weight bearing in both groups LCP: locking compression plate

Complete weight bearing (weeks)				
Weeks	Conventional LCP		Variable LCP	
	No.	Percentage (%)	No.	Percentage (%)
14-16	10	33.33	11	36.67
18-20	11	36.67	13	43.33
22-24	6	20.00	4	13.33
28-30	3	10.00	2	6.67
Total	30	100.00	30	100.00

**Figure 5:**
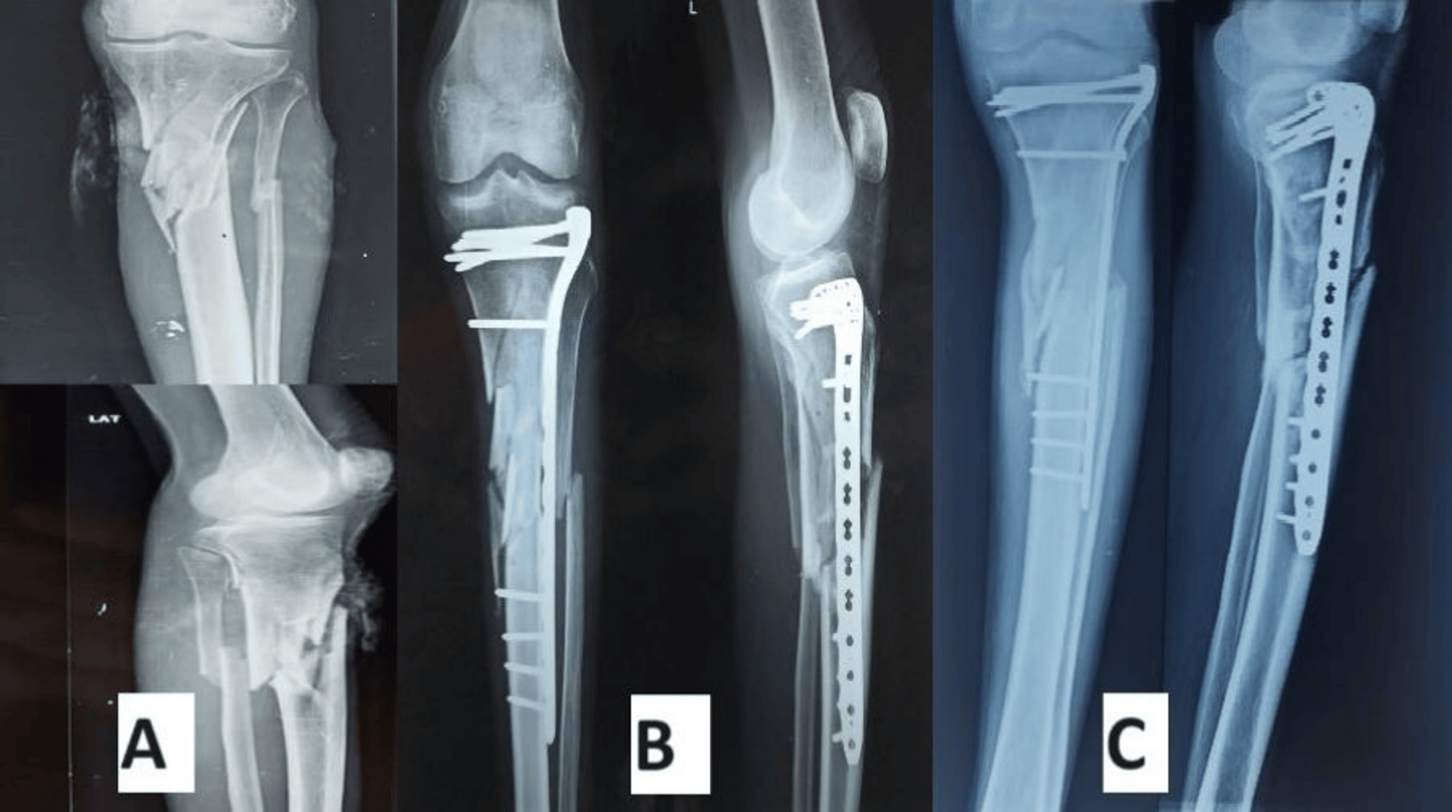
X-rays of the variable-angle locking plate group patient, 52-year-old male A) Preoperative X-rays showing tibial plateau fracture; B) Postoperative X-rays showing fracture fixed with variable-angle locking plate; C) Six months postoperative X-rays showing union of fracture

**Figure 6:**
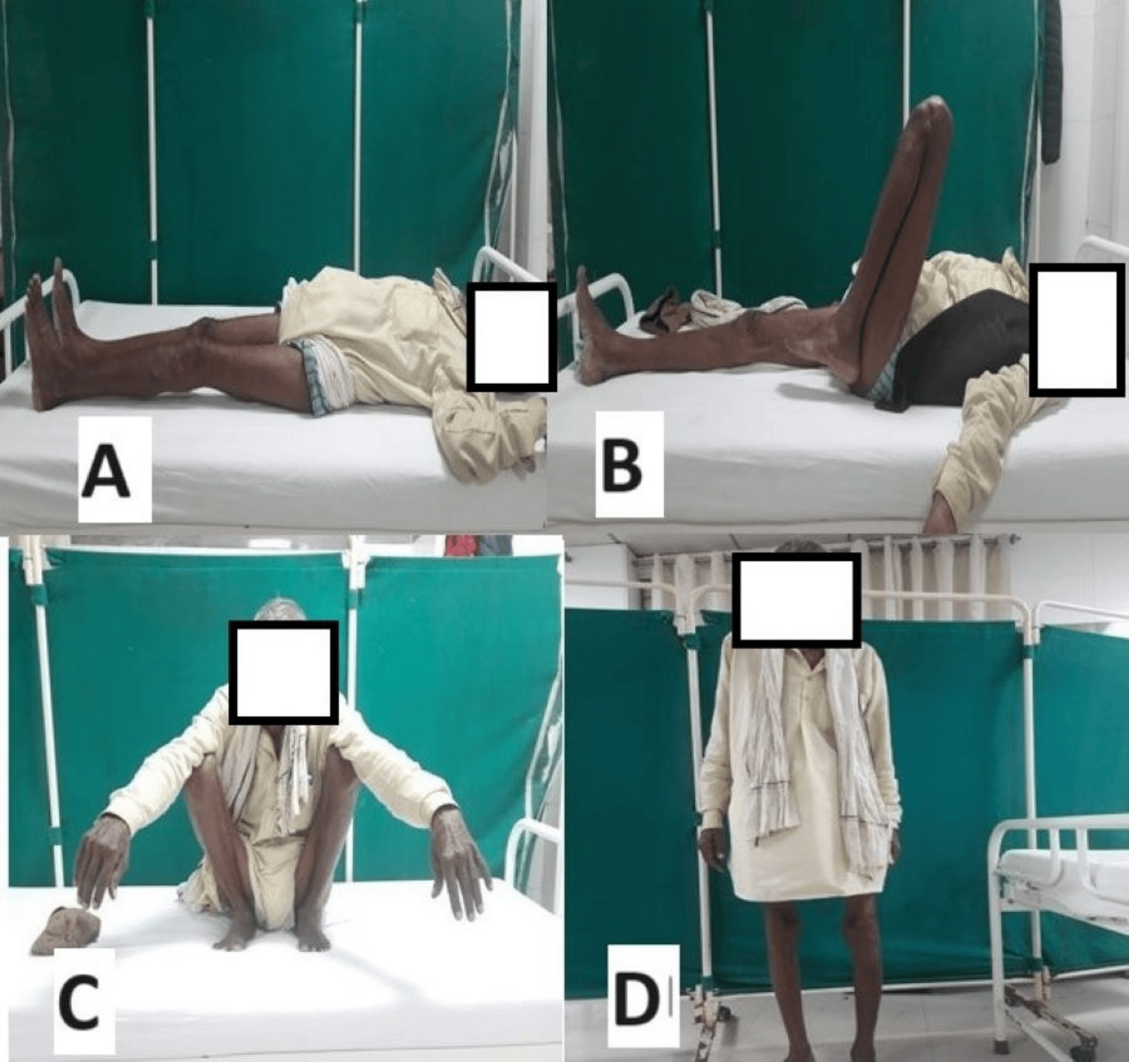
Clinical images of a 52-year-old male patient: variable angle locked plate group A) At final follow-up (six months) knee extension full (0 degree); B) At final follow-up (six months) knee flexion full (130 degree); C) At final follow-up (six months) patient squatting position; D) At final follow-up (six months) patient full weight bearing position

**Figure 7:**
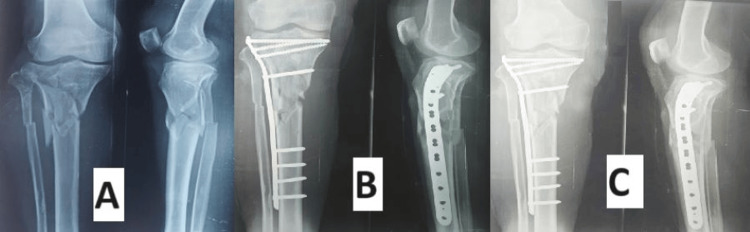
X-rays of conventional fixed angle locking plate group patient, 45-year-old male A) Preoperative X-rays of patient showing tibial plateau fracture; B) Postoperative X-rays of patient showing fracture fixed with fixed angle proximal tibia locking plate; C) Six months postoperative X-rays of patient showing union of fracture

**Figure 8:**
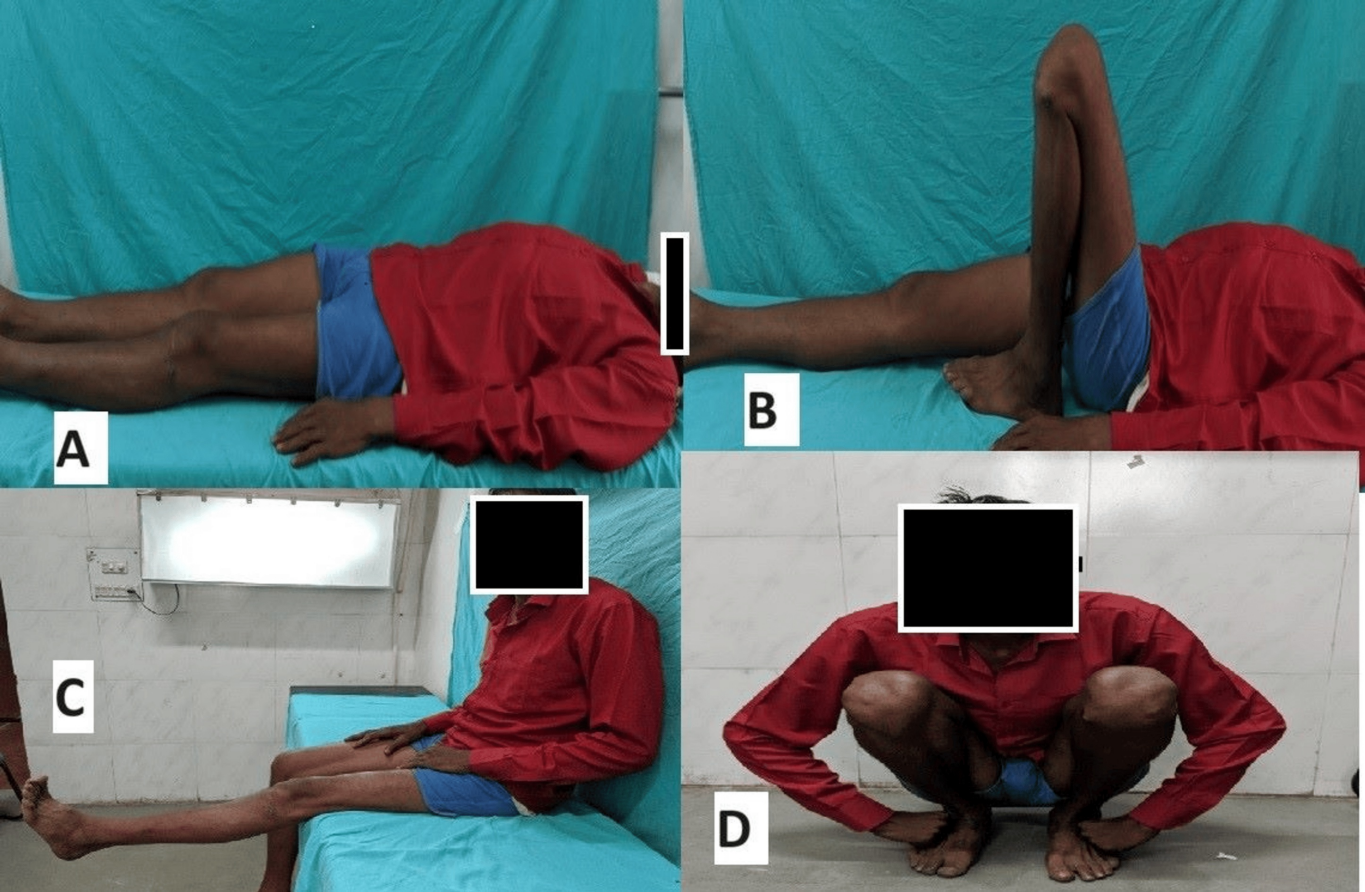
Clinical images of a 45-year-old male patient: conventional fixed-angle locking plate group A) At final follow-up (six months) full extension of operated left knee; B) At final follow up (six months) full flexion of operated left knee; C) At final follow up (six months) full extension of operated left knee with optimum quadriceps power; D) At final follow up (six months) patient in squatting position

According to Rasmussen's functional grading system at six months, in the VALCP group, 18 patients (60%) achieved excellent outcomes, nine patients (30%) had good results, two patients (6.67%) had fair results, and one patient (3.33%) had poor outcomes (Figures 5-6). In the conventional LCP group, 15 patients (50%) had excellent outcomes, 12 patients (40%) had good results, one patient (3.33%) had fair results, and two patients (6.66%) had poor outcomes (Figures 7-8). In all cases of both groups showed union at the fracture site on X-rays and radiological assessments using Rasmussen's grading system at final follow-up revealed that in the conventional LCP group, 22 patients (73.33%) had excellent results, and eight patients (26.67%) had good results, with no poor outcomes (Figures 5, 7). In the VALCP group, 24 patients (80%) achieved excellent results, five patients (16.67%) had good results, and one patient (3.33%) had fair results, with no poor outcomes. Based on the OKS, 26 patients (86.67%) in the VALCP group had excellent results, four patients (13.33%) had good results, and none had fair or poor outcomes. In the traditional LCP group, 22 patients (73.33%) achieved excellent outcomes, eight patients (26.67%) had good results, and no patients had fair or poor outcomes (Table 5, Figures 5-8).

**Table 5:** Functional results of both groups at final follow-up Unpaired student t-test was used as a test of significance. LCP: locking compression plate

Rasmussen’s functional grading system	Conventional LCP			Variable LCP			p-value
	No.	Percentage (%)	Mean±SD	No.	Percentage (%)	Mean±SD	0.389
Excellent	15	50.00	26.27±2.50	18	60.00	26.80±2.25	
Good	12	40.00		9	30.00		
Fair	1	3.33		2	6.67		
Poor	2	6.67		1	3.33		
Rasmussen’s radiological grading system							0.672
Excellent	22	73.33	9.07±0.87	24	80.00	9.17±0.95	
Good	8	26.67		5	16.67		
Fair	0	0.00		1	3.33		
Oxford Knee Score Sytem							0.129
Excellent	22	73.33	41.53±2.66	26	86.67	42.53±2.36	
Good	8	26.67		4	13.33		

Figures 5-6 show X-rays and clinical pictures of variable-angle locking plate group patients of a 53-year-old male having tibial plateau fracture while Figures 7-8 show radiological and clinical pictures of conventional fixed-angle locking plate group.

## Discussion

The management of tibial plateau fractures is complex and difficult to treat for a surgeon due to complex anatomy, fracture geometry, and complexities of the soft tissue envelope. With the development of infrastructure of highways and roads and with the advancement of high-speed vehicles in developing countries, the incidence of high-speed collisions, complex trauma, and fatal injuries is increasing drastically nowadays [11]. Reconstructing a stable painless mobile knee is a tough task and requires expertise and sufficient technical knowledge. With the advancement in imaging modalities and the introduction of modern instrumentation like locking plates, open reduction, and internal fixation became ideal management for this high-energy tibial plateau fractures [12].

The optimal classification system for proximal tibial fractures remains a subject of debate. In our study, patients were categorized using the Schatzker and AO classifications. Nevertheless, the mechanics of the accidents and related injuries are not adequately addressed by the Schatzkers classification; still, Schatzker and OTA/AO are most widely commonly utilized [13].

The treatment of proximal tibial fractures varies depending on the specific fracture type and the condition of the surrounding soft tissues. In our series, we utilized both conventional and variable-angle proximal tibia locking plates using the MIPPO technique in most cases. While most proximal tibial fractures require surgical intervention, uncomplicated and undisplaced fractures with minimal or no intra-articular involvement can be managed conservatively. Surgical treatment is often preferred because it not only facilitates morphological reduction but also enables early weight-bearing, immediate knee movement, and a faster return to pre-injury activities. Modern treatment methods frequently involve MIPO, employing newly designed locking plates [14].

For lateral unicondylar proximal tibia fractures (Schatzker's types I, II, and III), we utilized a single lateral locking plate, either with fixed or variable angles. We excluded medial condyle and bicondylar fractures (Schatzker's types IV, V, and VI) from the study because using a single lateral locking plate with screws extending through the plate to hold medial fragments in these cases is often associated with malunion. This can lead to varus deformity due to the collapse of the posteromedial fragment of the tibial plateau. For such fractures, medial plating or bicondylar plating is recommended to prevent these complications [15].

According to Yoo et al. (2010), laterally fastened angled plates are often ineffective at successfully securing screws into the posteromedial fragment (PMF). However, a study by Phillips et al. (2020) involving 114 bicondylar plateau fractures analyzed using CT scans suggests that modern plates, equipped with multiple rows of locking screws and variable angle technology, have improved the ability to capture the PMF. On average, these contemporary plates were able to secure 81.6% of the PMF with an average of 3.77 screws (95% confidence interval (CI): 3.47-4.07). Despite this, there was significant variability in the success of capturing all fragments, ranging from 55.7% to 95.2% in fixed-angle constructs. Notably, variable angle constructs demonstrated a significantly higher capture rate overall (98.5% vs. 74.9%) [16,17]. Variable-angle plating is introduced through the same incision. By capturing the fracture fragments in the desired direction, this technique reduces the risk of malunion and provides a strong fixation. This secure fixation enables early rehabilitation and allows for movement of the surrounding joints, helping to prevent joint stiffness [10,18,19].

In the present study, most of our cases were Type II Schatzker fractures, observed in 36 patients (60%), with the primary cause being traffic accidents, accounting for 37 patients (61.66%). Similarly, Shekhar and Pranjal (2022) reported that Type II fractures were the most common, occurring in 26 out of 54 patients (48.14%) [18]. Völk et al. (2021) found that 16 out of 28 patients (57.14%) had AO type 41B fractures, which correspond to Schatzker Type II and III fractures [19]. This pattern was also noted by Nikolaou et al. (2011) [10]. In all three studies, high-speed collisions were the leading cause of injury.

In our study, the average operating time for the traditional LCP group was 84 minutes, with a standard deviation of 7.71 minutes, compared to 81 minutes for the variable-angle LCP group, with a standard deviation of 7.28 minutes. This is consistent with findings by Völk et al. (2021), where the mean operative time was 150 minutes for the conventional LCP group and 130 minutes for the variable angle LCP group. These results suggest that traditional plating may be associated with a longer operative duration [19].

In our study, among the 30 patients treated with fixed LCP, 15 (50%) achieved excellent outcomes, 12 (40%) had good results, one (3.33%) had fair results, and two (6.67%) had poor results. In contrast, among the 30 patients with variable angle plating, 18 (60%) had excellent outcomes, nine (30%) had good results, two (6.67%) had fair results, and one (3.33%) had poor results. The mean Rasmussen functional score at the six-month follow-up was 26.80 for the variable angle LCP group and 26.27 for the traditional plating group, but this difference was not statistically significant. For the OKS, 26 (86.67%) variable-angle LCP patients had an average score of 43, indicating excellent results, while four (13.33%) had good results with an average score of 37.25; no patients had fair or poor results. Among the fixed-angle LCP patients, 22 (73.33%) had an average score of 42.67, reflecting excellent results, and eight (26.67%) had a score of 37.8, indicating good results; no patients had fair or poor outcomes. The mean OKS was 42.53 for the variable angle LCP group and 41.53 for the fixed angle LCP group, with no statistically significant difference found.

There is limited research specifically comparing fixed versus variable-angled proximal tibia plating. However, existing studies underscore the effectiveness of fixed and variable-angled fixation methods for tibial plateau fractures. A pioneering study by Nikolaou et al. (2010) utilized polyaxial locked plating in 60 patients with fractures classified as 41-A, 41-B, and 41-C. Their results consistently demonstrated the efficacy of this approach, aligning with our study's findings. They concluded that the polyaxial locking-plate system offered excellent functional results, a low complication rate, and stable repair for both intra- and extra-articular proximal tibial fractures. Another relevant study by Shekhar and Pranja (2022) involved 54 patients with Schatzker types I, II, and III proximal tibial fractures, all treated with VALCP. Their outcomes, assessed using modified Rasmussen clinical and radiological scores over a 24-week period, mirrored those observed in our study's variable-angle group. Additionally, Völk et al. (2021) conducted a comparative study with 28 patients, examining various polyaxial locking plates. They found that the variable-angle locking plate group had better results compared to the fixed-angle group, though the differences in clinical and radiological outcomes between the groups were not statistically significant. These findings support the results observed in our study [10,18,19].

The complications observed in our study were not as extensive as those reported in previous literature on locking plate fixation for proximal tibia fractures. A study by Khatri et al. (2016) found that superficial wound infection was the most common complication. In their analysis of 62 cases, the overall complication rate was 19.65%, with 20.97% of cases (13/62) involving infectious complications. Of these, eight patients experienced superficial infections, managed with regular dressing changes and antibiotics. Five patients who developed deep infections required more extensive interventions, including multiple debridements, flap coverage, implant removal, or amputation, depending on the severity of the infection and the patient's response [20].

## Conclusions

This prospective study included 30 patients per group with tibial plateau fractures (Schatzker types I to III), chosen randomly without predetermined protocols. The follow-up period was relatively short. Based on our analysis, VALCP plating appears to be a promising treatment for Schatzker types I, II, and III fractures, offering superior fixation, early mobilization, excellent to good functional and radiological outcomes, and fewer complications compared to conventional plating. However, there was no significant difference in functional or radiological outcomes between VALCP and conventional plating. Future research should involve larger, randomized trials to provide a more thorough comparison of conventional and VALCP locking plates for proximal tibia fractures. This study did not compare VALCP with other implant options or address bicondylar fractures (Schatzker types IV, V, and VI). Additionally, the limited number of patients and short study duration did not allow for an evaluation of long-term complications or revision surgeries. Future studies should be more comprehensive, well-designed, and adequately powered to address these gaps.
